# Lipid peroxidation of immune cells in cancer

**DOI:** 10.3389/fimmu.2023.1322746

**Published:** 2024-01-08

**Authors:** Liuling Xiao, Miao Xian, Chuanchao Zhang, Qi Guo, Qing Yi

**Affiliations:** Center for Translational Research in Hematologic Malignancies, Houston Methodist Neal Cancer Center, Houston Methodist Research Institute, Houston Methodist, Houston, TX, United States

**Keywords:** lipid peroxidation, TME, innate immune cells, adaptive immune cells, tumor immunity

## Abstract

Growing evidence indicates that cellular metabolism is a critical determinant of immune cell viability and function in antitumor immunity and lipid metabolism is important for immune cell activation and adaptation to the tumor microenvironment (TME). Lipid peroxidation is a process in which oxidants attack lipid-containing carbon-carbon double bonds and is an important part of lipid metabolism. In the past decades, studies have shown that lipid peroxidation participates in signal transduction to control cell proliferation, differentiation, and cell death, which is essential for cell function execution and human health. More importantly, recent studies have shown that lipid peroxidation affects immune cell function to modulate tumor immunity and antitumor ability. In this review, we briefly overview the effect of lipid peroxidation on the adaptive and innate immune cell activation and function in TME and discuss the effectiveness and sensitivity of the antitumor ability of immune cells by regulating lipid peroxidation.

## 1 Introduction

Lipid peroxidation is a complex process in which oxidants attack lipids-containing carbon-carbon double bonds, leading to oxidative degradation of lipids, formation of lipid peroxides, and subsequent cellular condition change (1). As an important aspect of lipid metabolism, lipid peroxidation is essential for the execution of cell function and human health (1–3). In recent years, accompanied by the discovery of ferroptosis that is a unique form of regulated cell death characterized by overwhelmed lipid hydroperoxides in cells (4, 5), lipid peroxidation gains increasing attention from researchers. For instance, interferon-γ (IFN-γ) secreted by activated CD8^+^ T cells in PD-L1 blockade therapy induced increased lipid peroxidation in tumor cells and contributed to the antitumor efficacy of immunotherapy (6). Administration of cyst(e)incase in pancreatic ductal adenocarcinoma mouse model resulted in an accumulation of lipid peroxides and consequent tumor regression (7). However, tumor develops in a complex and dynamic microenvironment that includes not only tumor cells, but also immune cells (8, 9). In TME, immune cells play important roles in tumorigenesis. They can secrete cytotoxic molecules to eliminate tumor cells, or they produce suppressive cytokines to drive chronic inflammation to stimulate cancer metastasis (8, 9). The accumulation of lipid peroxidation in TME affects both tumor and immune cells. Therefore, a comprehensive understanding of the role of lipid peroxidation in different cells in TME is essential for providing new targets and therapeutic approaches to treat cancers (10–12). Several excellent reviews have discussed the role of lipid peroxidation in tumor cells and can be referred to for details (10, 13, 14). In this review, we will focus on how lipid peroxidation mediates immune cell function and how this process affect tumor progression.

## 2 An overview of lipid peroxidation

**Figure 1:**
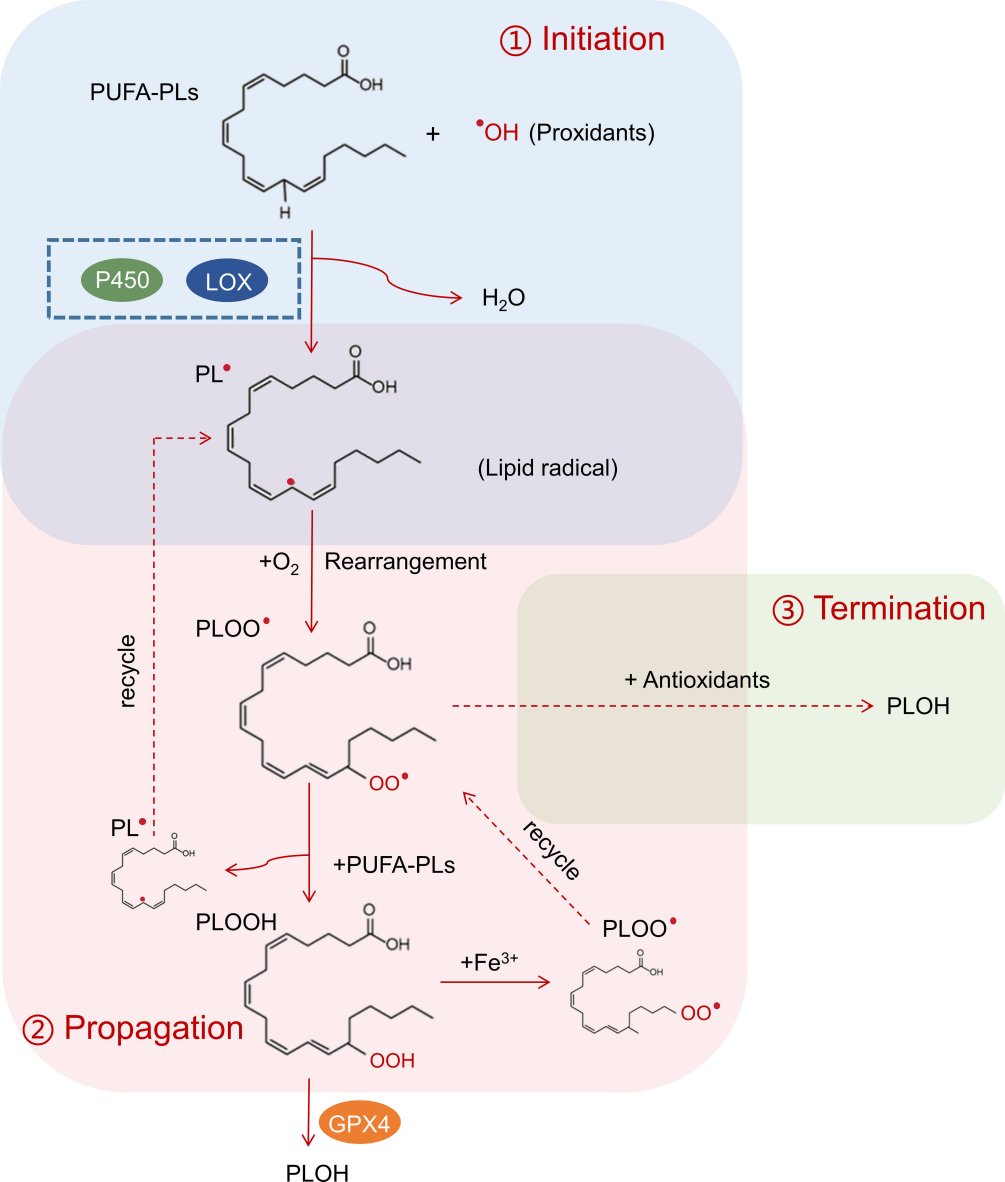
Process of lipid peroxidation. In the initiation process (blue box), interaction between PUFA-PLs and prooxidant hydroxyl radicals (•OH) leads to the formation of carbon-centered phospholipid radical (PL•), which can be catalyzed by lipoxygenases (LOX) and P450 oxidoreductase. In the propagation step (red box), phospholipid radical (PL•) rapidly reacts with oxygen and experiences an arrangement to form a phospholipid peroxy radical (PLOO•), which then abstracts a hydrogen from another PUFA-PL molecule to generate a new PL• and phospholipid hydroperoxide (PLOOH). The new PL• can directly recycle to the propagation step to create a chain reaction. GPX4 can convert highly active PLOOH to stabilize phospholipid alcohol (PLOH) to stop the chain reaction. In the termination phase (green box), antioxidants react with PLOO• and form PLOH.

Lipid peroxidation is a metabolic process that involves the oxidative degradation of lipids and consists of three stages: initiation, propagation, and termination (1, 11, 15) (**Figure 1**). The mechanisms of lipid peroxidation are still not fully understood and can be catalyzed by both enzymatic and non-enzymatic formats (1, 11). For the enzymatic format, lipoxygenases (LOX) and P450 oxidoreductase have been implicated in this process (11, 15). In the initiation stage, prooxidants such as hydroxyl radicals (•OH) or peroxyl radicals (ROO•) abstract the allylic hydrogen atom from polyunsaturated fatty acyl-containing phospholipids (PUFA-PL) to form the carbon-centered phospholipid radical (PL•). The allylic hydrogen atom is usually located between two carbon-carbon bonds, and this is why lipid peroxidation is more frequently associated with polyunsaturated fatty acids (PUFA) (11, 16). In the propagation step, phospholipid radical (PL•) rapidly reacts with oxygen and experiences an arrangement to form a phospholipid peroxy radical (PLOO•), which then abstracts a hydrogen from another PUFA-PL molecule to generate a new PL• and phospholipid hydroperoxide (PLOOH). The new PL• can directly recycle to the propagation step and PLOOH can interact with cellular labile iron to generate PLOO• to continue the chain reactions (17). If glutathione peroxidase 4 (GPX4) is present, it can convert highly active PLOOH to stabilized phospholipid alcohol (PLOH) (11, 18). In the termination phase, antioxidants like vitamin E provide a hydrogen atom to the PLOO• and form PLOH (19). The propagation phase is an amplified chain reaction and leads to the production of PLOOHs. If they are not terminated by antioxidants or GPX4, these lipid hydroperoxides (PLOOHs) are unstable and can eventually lead to the formation of secondary products such as malondialdehyde (MDA), 4-hydroxynonenal (4-HNE) and other reactive aldehydes (1, 20). Although appropriate lipid peroxidation plays important roles in physiological processes including cell signaling, immune responses, and apoptosis, excessive lipid peroxidation is associated with cellular oxidative stress and participates in numerous pathological conditions, such as inflammation, neurodegenerative disorders, cardiovascular diseases, and cancers (2, 3, 14, 20).

In the context of cancer, lipid peroxidation contributes to tumor progression by promoting genomic instability, activating pro-inflammatory pathways, and inducing immune evasion (1–3). Recent years have seen significant advancements in our understanding of how lipid peroxidation affects immune cells within TME. From innate to adaptive immunity, lipid peroxidation influences the activation, polarization, and function of various immune cells as we will discuss below.

## 3 Immune cell composition in TME

The immune system is critical for cancer initiation and progression. When a tumor develops, the host spontaneously elicits an immune response to suppress the growth of tumor cells (21). To survive and expand *in vivo*, tumor cells have developed various methods to avoid the recognition and attack by the immune system, which is called immune evasion (22). Therefore, interactions between tumor cells and immune cells during tumor growth are divided into three stages: elimination, equilibrium, and evasion (22). As studies on TME and cancer immunotherapy accumulate, researchers found that TME comprises a range of distinct immune cells arising from the innate and adaptive immune systems.

In the adaptive immune system, there are T cells (CD4^+^, CD8^+^ T, and NKT cells), B cells, and plasma cells in TME. CD8^+^ T cells are the most well-studied antitumor effect cells that can directly kill tumor cells by cytotoxic cytokines and induction of apoptosis. CD4^+^ T cells comprise several subsets including T helper 1 (Th1) (23), T helper 2 (Th2) (24), T helper 9 (Th9) (25, 26), T helper 17 (Th17) (27) and regulatory T (Treg) cells (28). The functions of each CD4^+^ T cell subset are different. Th1 and Th9 cells are antitumor effector cells, while Treg are well-known immunosuppressive cells that reduce the effectiveness of antitumor effector cells and promote tumor progression. As for NKT cells, they can exert either antitumor or immunosuppressive abilities depending on their TCR activation and immunologic context. Relative to T cells, B cells and plasma cells are less well understood and their functions on TME are being explored.

The innate immune system, including natural killer (NK) cells, tumor-associated macrophages (TAM), myeloid-derived suppressor cells (MDSC), dendritic cells (DCs), and granulocytes, acts as the body’s first line of defense and bridge to adaptive immunity (29–31). Similar to CD8^+^ T cells, NK cells are critical effector cells to control tumor growth in both hematologic malignancies and solid tumors. However, MDSC are well-known immunosuppressive cells in TME. Interestingly, TAM is divided into two classes, inflammatory TAM and immunosuppressive TAM, which suppress or promote tumor progression respectively. In line with TAM, DCs are a heterogeneous hematopoietic lineage with various subsets including conventional DCs (cDCs) and plasmacytoid DCs (pDCs). Although DCs are the most potent professional antigen-presenting cells, they can stimulate T cell activation to suppress tumor cell growth or become tolerant in certain TME to exhibit immunosuppressive properties. Lastly, granulocytes, including neutrophils, eosinophils, basophils, and mast cells, are complex and have distinct functions in different tumor stages.

Although there are various immune cells in TME, it has become increasingly clear that their functions are affected by metabolism conditions. Immune cells engage in a specific metabolic program to maintain their function and activity. However, highly active tumor cells can create profound changes in nutrient, oxygen concentration, and acidity in TME that affect the metabolism program in immune cells and eventually achieve immune evasion. Accumulating evidence suggests that altering immune cell metabolism is a promising approach to enhance cancer immunotherapy, and lipid peroxidation is a potential target to fulfill this goal. Therefore, we will discuss the role of lipid peroxidation in different immune cells in the following sections.

## 4 The effect of lipid peroxidation on immune cells in cancer

### 4.1 The impact of lipid peroxidation on adaptive immunity

**Figure 2:**
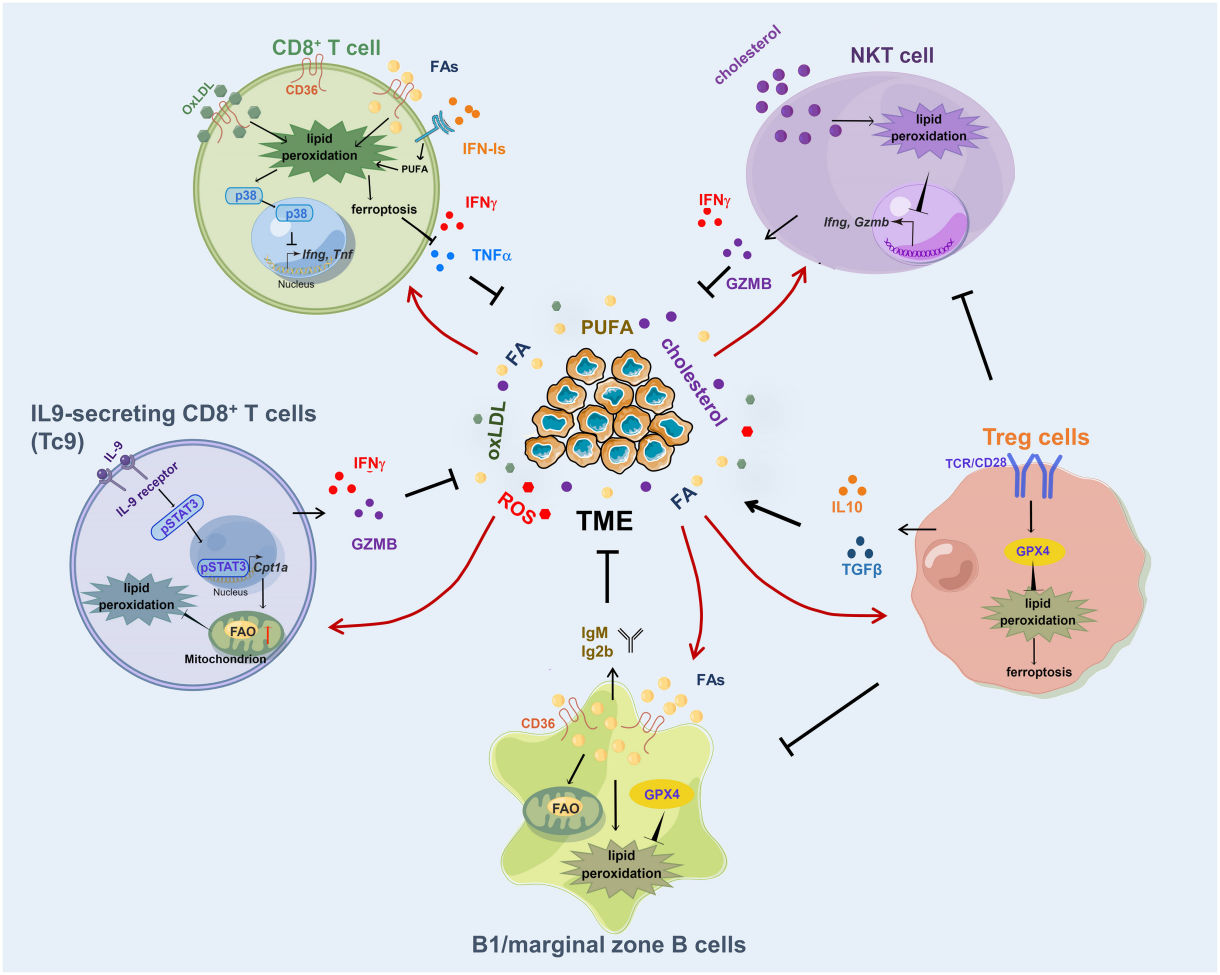
Lipid peroxidation in adaptive immune cells. Excessive fatty acid or OxLDL uptake through CD36 induces an increased lipid peroxidation in CD8^+^ T cells, resulting in ferroptosis and impaired production of cytotoxic cytokines. IL-9 activates STAT3 to upregulate fatty acid oxidation and mitochondrial activity in Tc9 cells and renders the cells with reduced lipid peroxidation and ferroptosis. GPX4 is required for Treg cells to clear lipid peroxides produced in antigen activation process. Cholesterol induces lipid peroxidation and decreases cytotoxicity of NKT cells. B1 and marginal zone B cells require GPX4 to prevent lipid peroxidation in response to increased fatty acid uptake and lipid droplet storage. FA, fatty acid; OxLDL, oxidized low-density lipoproteins.

Adaptive immunity is characterized by its ability to recognize and specifically target particular antigens and plays a significant role in the body’s response to cancer. The major adaptive immune cells include T cells and B cells, and the function of adaptive immune cells is affected by lipid peroxidation (**Figure 2**).

#### 4.1.1 Lipid peroxidation of T cells

T cells are critical components of the adaptive immune system. When naïve T cells recognize their cognate antigens by T cell receptor (TCR), they become activated and proliferative with the support of co-stimulatory signal (32). It has been reported that the lipid peroxidation products 4-HNE and MDA can impair T cell activation by the TCR signaling pathway (33, 34). Upon TCR activation, T cells would induce the production of hydrogen peroxide or superoxide anion in cellular (35) If cellular lipid peroxidation is not scavenged by GPX4 or antioxidants, rapidly accumulated membrane lipid peroxides in T cells would induce ferroptosis and suppress T cell immunity (36). In follicular helper T cells, there is an intensified lipid peroxidation, and GPX4 is the major lipid peroxidation scavenger to support follicular helper T cell survival (37).

##### 4.1.1.1 CD8^+^ T cells

Different from tissue-resident T cells, tumor-infiltrating T cells possess unique metabolic features (8). A recent study showed that CD8^+^ T cells derived from tumors displayed higher levels of lipid peroxidation than those from lymph nodes, and they were sensitive to GPX4 inhibitors-induced ferroptosis (38). Consistent with this result, other groups found that the lipid peroxidation accumulation in tumor-infiltrating CD8^+^ T cells was the result of excessive uptake of PUFA or oxidized lipid (39, 40). Different from PUFA, short-chain fatty acids (SCFAs) are less susceptible to lipid peroxidation and have different effects on T cells. SCFAs pentanoate and butyrate were discovered to increase the function of mTOR and inhibit class I histone deacetylase activity, which leads to elevated production of effector molecules such as CD25, IFN-γ, and TNF-α, and significantly enhances the anti-tumor activity of T cells (41). Moreover, fatty acid oxidation of certain microbiota-derived SCFAs promoted the differentiation of effector CD8^+^ T cells with memory potential and enhanced recall capacity (42). As the most powerful effectors in the adaptive immune system to suppress tumor progression, tumor-infiltrating CD8^+^ T cells utilize fatty acids as an alternative energy to maintain their function in the glucose-deprived TME (8, 43, 44). However, excessive fatty acid uptake through CD36 can induce lipid peroxidation accumulation in CD8^+^ T cells (45). Increased lipid peroxidation promoted CD8^+^ T cell ferroptosis, and impaired their cytotoxic cytokines production and antitumor ability (45). Likewise, CD36 also uptake oxidized lipid to upregulate lipid peroxidation in CD8^+^ T cells and activate the p38 kinase pathway to decrease the transcription of effector cytokine genes, leading to T cell exhaustion and tumor progression (40). Decreasing cellular lipid peroxidation by depleting CD36 or overexpressing GPX4 in CD8^+^ T cells rescued the function of T cells and achieved enhanced antitumor ability (40, 45). Besides lipids, TME is often enriched with inflammatory cytokines, and tumor-infiltrating CD8^+^ T cells experience persistent type-I IFN (IFN-I) signaling (46). Chronic IFN-I stimulation perturbs lipid metabolism and redox balance in exhausted CD8^+^ T cells, leading to aberrant lipid accumulation and elevated lipid peroxidation, which exaggerates metabolic and functional exhaustion of T cells (46).

##### 4.1.1.2 IL9-secreting CD8^+^ T cells

Besides conventional CD8^+^ T cells, there is a specific subset of CD8^+^ T cells named Tc9 cells, which is generated by differentiating naïve CD8^+^ T cells in Th9-polarizing medium, that resists reactive oxygen species (ROS)-induced lipid peroxidation in TME (47–49). Tc9 cell-derived IL9 activated STAT3 in an autocrine manner, upregulated fatty acid oxidation (FAO) and mitochondrial activity, and rendered Tc9 cells with reduced lipid peroxidation and ferroptosis in TME, leading to longevity and enhanced antitumor activity of adoptively transferred Tc9 cells *in vivo (* 49). This finding is consistent with previous studies that FAO was a key requirement for T cell development as modulating FAO *in vivo* would enhance the generation of CD8^+^ T memory cells (50) and increasing FAO and mitochondrial respiratory capacity by PGC-1α/PPAR complexes can support the survival of T cells (51). In addition, treating mice with the agonist of the PPAR signaling pathway has a synergistic effect with anti-PD-1 therapy (51).

##### 4.1.1.3 Treg cells

Different from CD8^+^ T cells, studies have shown that intratumoral Treg cells were adapted to lipid-enriched and oxidative-stressed TME (39, 52, 53). In Treg cells, increased CD36 expression is necessary for mitochondrial fitness through peroxisome proliferator-activated receptor-β signaling and programs Treg cells to adapt to a lactic acid-enriched TME (39). Upon activation by antigens in TME, GPX4 was required for Treg to clear lipid peroxides produced in the activation and expansion process to support their function (54–56). When *Gpx4* was deficient, excessive lipid peroxides were accumulated in Treg cells in response to TCR and co-stimulatory signals, followed by elevated production of mitochondrial superoxide and IL1β and conversion to Th17-type responses (56). Moreover, the depletion of Gpx4 in Treg cells suppressed tumor growth and enhanced antitumor immunity. However, whether and how GPX4 is highly expressed in Treg cells other than CD8^+^ T cells is still unknown.

##### 4.1.1.4 NKT cells

NKT cells are a subset of CD1d-restricted T cells that have properties of both T cells and natural killer cells, and bridge the innate and adaptive immune systems (57). CD1d molecule is a non-classical MHC protein that presents lipid and glycolipid antigens to T cells (58). Although the number of NKT cells in peripheral blood is low, they are enriched in liver, account for 30–40% and 5–25% of liver lymphocytes in mouse and human respectively, and play important roles in suppressing the development of hepatocellular carcinoma (59). Interestingly, the antitumor ability of NKT cells was impaired by cellular lipid peroxidation accumulation (60). In obesogenic-diet-promoted hepatocellular carcinoma, excessive cholesterol production from hepatocytes impaired NKT expansion and cytotoxicity by lipid peroxide accumulation, resulting in a diminished antitumor response (60). However, the underlying mechanism of how lipid peroxidation affects the function of NKT needs further study.

Although tumor-infiltrating T cells adapted their metabolic program in lipid-enriched TME to harness lipids for energy to overcome the shortage of glucose, excessive lipid uptake, particularly oxidized lipids, can lead to the accumulation of lipid peroxidation in T cells. This accumulation has the potential to skew T cell differentiation and promote T cell dysfunction and death. Enhancing the lipid peroxidation clearance capability of effector CD8^+^ T cells or adoptively transferring lipid peroxidation-resistant CD8^+^ T cells may be promising strategies to enhance the effectiveness of cancer immunotherapy.

#### 4.1.2 Lipid peroxidation and B cells

Although much of the immunotherapeutic strategies have been focused on T cell-mediated immunity, accumulative evidence shows that B cells also play crucial roles in modulating tumor responses (61). Similar to T cells, the function of B cells is mediated by lipid metabolism as well (62). For example, it was reported that 4-HNE, the end-product of lipid peroxidation, induced apoptosis in human leukemic B cell lines (63). In primary B1 and marginal zone B cells, they required Gpx4 to prevent lipid peroxidation in the case of increased fatty acid synthesis, lipid uptake, and lipid droplet storage (64). When Gpx4 was deficient, there was increased lipid peroxidation and impaired antibody responses in B1 and marginal zone B cells (64), which may explain why GPX4-positive patients had poor overall survival than GPX4-negative patients in B-cell lymphoma (65).

### 4.2 The role of lipid peroxidation in innate immune cells

**Figure 3:**
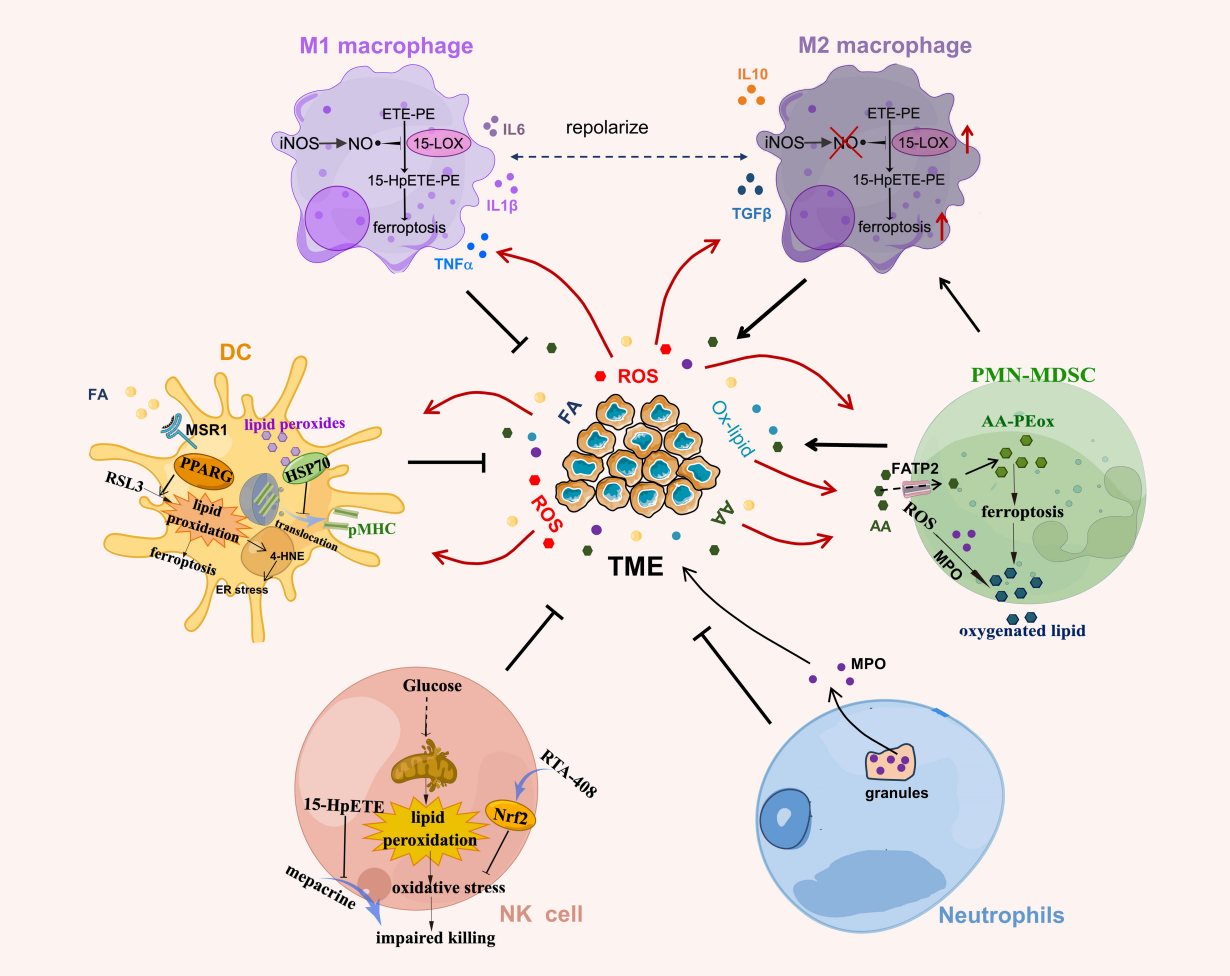
Lipid peroxidation in innate immune cells. NO generated by iNOS interacts with lipid peroxides to reduce the production of lipid peroxide 15-HpETE-PE that is catalyzed by 15-LOX in M1 macrophages. Lipid peroxides in DCs covalently bind to heat shock protein (HSP)70 and prevent the translocation of pMHC from late endosomes/lysosomes to cell surface. PPARγ promotes RSL3-induced lipid peroxidation and ferroptosis in DCs. The lipid peroxide 15-hydroperoxyeicosatetraenoic acid (Hp-ETE) reverses mepacrine-induced inhibition of human NK cell cytotoxicity and lipid peroxidation-associated oxidative stress induced by altered glucose metabolism induces NK cell dysfunction. Activation of antioxidant Nrf2 signaling by RTA-408 reverses this process. PMN-MDSC produce high levels of ROS and myeloperoxidase (MPO) to induce lipid peroxidation in PMN-MDSC, which spontaneously undergo ferroptosis due to increased lipid peroxides (AA-PEox) in TME. AA, arachidonic acid; Ox-lipid, oxidized lipid.

As innate immune responses are critical for controlling tumor progression, tumor cells take various actions to alter the function of innate immune cells to achieve immune evasion. In line with adaptive immune cells, the development and function of innate immune cells are modulated by cellular lipid peroxidation as well (**Figure 3**).

#### 4.2.1 Lipid peroxidation and macrophage

Macrophages are an important component of the immune system and can be polarized into classically activated macrophages (M1) or alternatively activated macrophages (M2) in response to microenvironmental signals (66). In general, M1 macrophages suppress tumor invasion by secreting proinflammatory cytokines whereas M2 macrophages promote tumor progression by secreting anti-inflammatory cytokines (66). Emerging evidence suggests that TAM predominantly exhibits the immunosuppressive M2-like phenotype and has increased lipid accumulation in TME by upregulating CD36 (67, 68). These changes shape them with immunosuppressive phenotype and promote the development of multiple myeloma and liver metastasis (67, 68).

As macrophages are phagocytic immune cells and usually produce ROS in digesting dying cells or microorganisms (69), it is possible that increased lipid content in macrophages induces cellular lipid peroxidation accumulation. Indeed, a recent study showed that lipid-enriched M2 macrophages had a higher level of lipid peroxide products 15-HpETE-PE than M1 macrophages (70). 15-HpETE-PE is catalyzed by the 15-lipoxygenase (15-LOX), which is encoded by *Alox5* gene and can oxygenate polyunsaturated fatty acids to lipid peroxides (71). M1 macrophages had a lower expression of 15-LOX and were enriched with iNOS/NO•products. NO• generated by iNOS could interact with lipid peroxides and secondary lipid radical intermediates catalyzed by 15-LOX (70) and substitute GPX4 as an anti-ferroptotic defense, which renders M1 macrophages with high resistance to pharmacologically induced ferroptosis. Interestingly, when transferring iNOS deficient *(Nos2* ^-/-^) or wild-type bone marrow cells into irradiated recipient mice followed by Lewis lung carcinoma establishment, mice reconstituted with NOS2-deficient bone marrow cells had fewer M1 and similar numbers of M2 macrophages than mice reconstituted with bone marrow from wild-type mice. These results suggest that iNOS is required for M1 macrophage survival in ROS-enriched TME. Unexpectedly, tumor burden was significantly reduced in mice with NOS2-deficient bone marrow chimers compared to the wild-type mice, suggesting that even though the number of M2 macrophages in iNOS-deficient TME is not affected, the immunosuppressive function of M2 macrophages may change. In another study, Hsu et al. reported that lipid peroxidation product 4-HNE inhibited pyroptosis and inflammasome activation of LPS stimulated macrophages (M1 macrophage) by directly disrupting the binding of NIMA-related kinases 7 to NLR Family Pyrin Domain Containing 3 (NLRP3) (72). In support of this result, Kapralov et al. showed that deletion of GPX4 inhibits the survival of M2 other than M1 macrophages (70). Those studies highlighted the role of lipid peroxidation on macrophage function and survival. As the sensitivity of M1 and M2 macrophages to lipid peroxidation products are different, targeting macrophages with lipid peroxidation inducer to eliminate M2 macrophages in TME is a potentially promising strategy to improve cancer immunotherapy efficacy, and more investigations are needed to test this hypothesis.

Different cytokines secreted by macrophages also have distinct effects on lipid peroxidation. Classic M1 macrophages mainly secrete pro-inflammatory factors like TNFα, IL1α, IL1β, IL6, IL12 and IL23. For M2 macrophages, some subtypes of M2 macrophages (M2a, M2c) produce anti-inflammatory factors such as IL10 and TGFβ, while some subtype (M2b) secrete TNFα, IL1β, IL6 and IL10 (73). Among the M1-secreted cytokines, IL6 can induce lipid peroxidation and disrupt iron homeostasis in bronchial epithelial cells, ultimately resulting in ferroptotosis (74). TNFα induces the expression of ACSL3 (75), which is required for exogenous monounsaturated fatty acids to block lipid peroxidation accumulation on the plasma membrane (76). IL1β increased lipid peroxidation in rat hippocampal tissue (77), and intraperitoneal injection of IL1β could induce accumulation of the end-products of lipid peroxidation in all structures of the brain in rats (78). As for cytokines from M2, a very recent study found that IL10 protects oligodendrocyte progenitor cells from ferroptosis by modulating the STAT3/DLK1/ACC axis to decrease the accumulation of lipid peroxidation (79). On the other hand, TGFβ, another cytokine secreted by M2 macrophages, was found to provoke redox imbalance and directly induced lipid peroxidation in hepatocellular carcinoma cells (80).

Innate immune responses are characterized by the initial influx of polymorphonuclear leukocytes, followed by monocyte-derived macrophages, ultimately leading to tissue resolution and the restoration of normal physiology (81). During this process, a crucial shift in the lipid class occurs—from pro-inflammatory lipid mediators derived from arachidonic acid to anti-inflammatory lipid mediators derived from docosahexaenoic acid (DHA) and eicosapentaenoic acid (EPA). This transition plays a pivotal role in the resolution phase (81). The acute response initiation involves changes in blood flow stimulated by prostaglandin E2 and prostaglandin I2, while leukotriene B4, produced from arachidonic acid, stimulates the recruitment of polymorphonuclear leukocytes. In later stages, the ω-3 essential fatty acids EPA and DHA are identified as substrates for the biosynthesis of potent anti-inflammatory and pro-resolving endogenous mediators within inflammatory resolving exudates (82). Notably, resolvins counterregulate the release of cytokines/chemokines, thereby suppressing tumor growth and enhancing cancer therapy (83). Given that arachidonic acid, DHA, and EPA are all PUFAs with the potential for lipid peroxidation, further investigation into the role of lipid peroxidation in the resolution process of macrophages is needed.

#### 4.2.2 Lipid peroxidation and DCs

As professional antigen-presenting cells, DCs were reported to exhibit increased lipid accumulation in tumor-bearing mice and cancer patients (84, 85), which was caused by increased uptake of extracellular fatty acids through macrophage scavenger receptor A (MSR1) and impaired antigens presenting ability of DCs. Additionally, in the lung cancer patients, elevated intracellular lipid accumulation occurs in the form of triglycerides, and antitumor immunity of DCs is suppressed (85). Similar to CD36, MRS1 belongs to the scavenger receptor family and is critical for intracellular lipid transport and uptake (86). Moreover, proliferator-activated receptor γ (PPARγ), the master lipid metabolism regulator (87), can regulate RSL3-induced lipid peroxidation and ferroptosis in DCs. Genetic depletion of PPARγ efficiently restored the function of DCs and improved the antitumor ability of DCs vaccines (88), however, this study did not explore the underlying mechanism and another research showed that sustained PPARγ activation reduced the expression of costimulatory molecules and IL-12 in DCs, ultimately impairing their capacity to prime naïve CD4^+^ T cells (89). In addition, it was reported that the main composition of lipids accumulated in tumor supernatant-treated DCs was poly-unsaturated fatty acids (90). Since unsaturated fatty acids are highly susceptible to oxidation and are the main substrate of lipid peroxidation (1), DCs from tumor-bearing mice had a significantly higher level of lipid peroxides, such as oxidized linoleic acid and arachidonic acid (90).

Lipid peroxidation is a double-edged sword for the function of DCs. Proper lipid peroxide is essential for DCs maturation. Rothe et al. reported that 12/15-lipoxygenase (LOX) mediated enzymatic lipid oxidation regulates DCs activation and corresponding consecutive T cell responses (91). 12/15-LOX is encoded by *Alox15* and represents the murine ortholog of human 15-LOX (92). During the DCs maturation process, the expression of 12/15-LOX was gradually downregulated and determined the threshold of DCs activation via the generation of phospholipid oxidation products. Deletion of the *Alox15* in DCs accelerated maturation and aggravated T cell–dependent autoimmune responses (91). Leakage of endosomal antigens into the cytosol is a crucial step in the process of cross-presentation in DCs. Elevated production of reactive oxygen species (ROS) and lipid peroxidation induces the escape of antigens from endosomes to the cytosol, facilitating their degradation and promoting antigen presentation by DCs (93). Consistent with this study, it was observed that increased cellular lipid bodies enhance the antigen cross-presentation of human cDC2s (94), however, whether this process is related to the accumulation of lipid peroxidation remains unclear. Moreover, dysregulation of redox homeostasis induced lipid peroxidation in post-synaptic (DCs), and this was associated with increased efficiency in cross-presentation to CD8^+^ T cells (95).

However, overwhelming lipid peroxidation impaired the cross-presentation and antitumor ability of DCs (90). Mechanistically, oxidatively truncated lipids can covalently bind to heat shock protein 70 and prevent translocation of pMHC from late endosomes/lysosomes to the cell surface (96). In addition, lipid peroxidation byproducts 4-HNE in DCs would activate X-box binding protein 1 (XBP1) to induce ER stress, which eventually resulted in the inability of DCs to present tumor antigens and stimulate CD8^+^ T cell responses (97). Accordingly, DC-specific XBP1 deletion enhanced the infiltrating of effector T cells in tumors and extended host survival (97). Consistent with this result, the knockdown of XBP1 in CD8^+^ T cells also restored their antitumor activity in lipid laden and pro-exhausted TME (98). Therefore, blocking XBP1 or relieving ER stress to improve abnormal lipid or lipid peroxidation accumulation in immune cells may provide a novel approach to enhance the efficacy of cancer immunotherapy.

#### 4.2.3 Lipid peroxidation and NK cells

NK cells can directly kill cancer cells without prior sensitization and play a critical role in the innate immune response against tumors, however, the cytotoxicity of NK cells was impaired in immunosuppressive TME (99). In hepatic metastasis mice, restraint stress increased the content of lipid peroxidation products in liver tissue, which impaired NK cell activity (100). Although this study did not explore the mechanism of how lipid peroxidation impaired NK cell activity, Poznanski et al. recently reported that lipid peroxidation induced glucose metabolism suppression in human NK cells, resulting in NK cell dysfunction and decreased antitumor ability in a nutrient-deprived TME (101). Activation of the antioxidant Nrf2 signaling by RTA-408 can effectively decrease the expression of lipid peroxidation-associated proteins, restore glucose metabolism and cytotoxicity of NK cells, and enhance their antitumor activity (101).

In contrast, other studies reported that hydroxyl radical-mediated lipid peroxidation is required for NK cells to deliver cytotoxic factors (102). In addition, 15S-hydroperoxyeicosatetraenoic acid, the product of 15-LOX catalytic lipid peroxide, effectively reversed mepacrine-induced inhibition of human NK cell cytotoxicity against K562 cells (103). These controversial studies indicate that the function of NK cells is finely regulated by cellular lipid peroxidation and the role of lipid peroxidation on NK cells is not yet fully understood.

#### 4.2.4 Lipid peroxidation on neutrophils and PMN-MDSC

Neutrophils are a kind of granulocytes and are featured as lobulated nuclei and azurophilic granules, which are known to release reactive oxygen species during activation (104). About 40 years ago, Claster et al. observed that activated neutrophils could induce lipid peroxidation in human red cells, which may contribute to the pathogenesis of hemolysis-associated infectious diseases and hemoglobin can protect the cell from peroxidative damage (105). Thereafter, it was reported that neutrophil infiltration and related lipid peroxidation accumulation were associated with the process of gastric reperfusion injury (106), Crohn’s disease (107), COVID-19-associated tissue damage (108), and tumor progression (109). In a glioblastoma mouse model, neutrophils can transfer granules containing myeloperoxidase to tumor cells to induce lipid peroxide accumulation and ferroptosis in tumor cells, leading to tumor cell necrosis and glioblastoma progression (109).

Pathologically activated neutrophils are the main immunosuppressive immune cells in TME and are termed polymorphonuclear MDSC (PMN-MDSC). PMN-MDSC exclusively upregulates the expression of fatty acid transport protein 2 (FATP2) that uptake of arachidonic acid and oxidized lipid, which react with the reactive oxygen species and myeloperoxidase to induce the accumulation of lipid peroxidation in PMN-MDSC (110, 111). PMN-MDSC then transferred oxidative lipids to DCs to limit their cross-presentation ability and promote tumor progression (111). Moreover, in a recent study, Kim et al. reported that PMN-MDSC would spontaneously undergo ferroptosis due to increased lipid peroxidation in TME and release oxygenated lipids to limit T cell activity in both mouse model and humans. Inhibition of lipid peroxidation in PMN-MDSC in immunocompetent mice unexpectedly abrogated their suppressive activity and reduced tumor progression to synergize with immune checkpoint blockade to suppress tumor growth (112). MDSC upregulated the expression of system Xc- and neutral -acylsphingosine amidohydrolase (ASAH2) to avoid the excessive lipid peroxidation accumulation in the cells. This study proposed a new method of treating cancers by pharmacological induction of lipid peroxidation and ferroptosis in PMN-MDSC and revealed the complex nature of lipid peroxidation in mediating tumor immunity.

Taken together, cellular lipid peroxidation level is critical for modulating immune cell function and tumor immunity. On one hand, increased lipid peroxidation impairs cytotoxic cytokine secretion of CD8^+^ T cells and NKT cells, and decreases antibody production by B cells and antigen cross-presentation by DCs. On the other hand, Treg cells are adapted to lipid peroxide-enriched TME, and tumoral PMN-MDSC release oxygenated lipids to limit T cell activity to promote tumor growth. These studies demonstrate that each immune cell has different responses and sensitivity to lipid peroxidation in TME. Therefore, the complex nature of lipid peroxidation in TME must be considered when targeting lipid peroxidation to provide potential therapeutic for cancers.

## 5 Regulator of lipid peroxidation in tumor microenvironment

Lipid peroxidation in the TME is a complex process influenced by various factors. For example, numerous studies have demonstrated that during tumor progression, the increased metabolic activity of cancer cells and infiltrating immune cells can lead to elevated ROS levels. These ROS can initiate lipid peroxidation by abstracting hydrogen atoms from unsaturated fatty acids in cell membranes. With the emergence of new methods and ongoing advancement in research, multiple factors have been discovered to be involved in the regulation of lipid peroxidation in TME.

### 5.1 PFUAs in tumor microenvironment induce lipid peroxidation

PUFA is a type of dietary fat that contains more than one unsaturated carbon-carbon double bond in its chemical structure (113). Due to the presence of multiple double bonds in their molecular structure, PUFAs exhibit heightened susceptibility to oxidation, rendering them a primary target for lipid peroxidation (114, 115).

Many investigations have shown PUFA could induce lipid peroxidation in TME. In multiple myeloma patients, PUFA, particularly arachidonic, was notably decreased as the disease advanced through its stages (116). Subsequent *in vitro* experiments showed that the arachidonic acid-triggered cell death in multiple myeloma cell lines could be reversed by lipid peroxidation inhibitors (116, 117). Interestingly, arachidonic acid is also a ligand of PPARs (118) and can inhibit the growth of lung cancer cells by activating PPARγ and lipid peroxidation (119, 120). PPARγ antagonist (GW9662) was shown to decrease cellular sensitivity to lipid peroxidation-induced ferroptosis (121). However, overexpression of PPARα suppressed erastin-induced lipid peroxidation-related ferroptosis, indicating that the effect of PPAR members on lipid peroxidation may vary. Other PUFAs like DHA are also able to promote ferroptosis by increasing intracellular lipid peroxidation (122). For example, incubation of DHA elevated lipid peroxidation and paracellular permeability in caco-2 colon adenocarcinoma cells (123). Additionally, PPARγ was identified as a component activated by DHA to impair lymphoproliferative stimulation capacity of DCs (124). In breast cancer, acyl-CoA synthetase long-chain family member 4 (ACSL4) catalyzed the esterification of arachidonic acid and adrenic acid and promoted the formation of lipid peroxides (125). Integrated bioinformatics analysis further revealed that elevated levels of arachidonic acid metabolism could serve as a potential biomarker for favorable prognostic outcomes in breast cancer (126). Moreover, arachidonic acid possesses the capacity to synergize with IFN-γ derived from T cells, thereby instigating immunogenic ferroptosis within TME (127).

However, these results contradicted other studies suggesting that TME have elevated level of free fatty acid (45, 128). In these studies, researchers found that in mouse B16 melanoma and Vk*MYC multiple myeloma models, the contents of fatty acids in tumor tissues were higher than in normal tissues. Fatty acid, particularly arachidonic acid, significantly increased lipid peroxidation and ferroptosis of CD8^+^ T cells (45). Similarly, the accumulation of long-chain fatty acids in TME of pancreatic ductal adenocarcinoma drives lipotoxicity in tumor-infiltrating CD8^+^ T cells (128). Although the detection of fatty acid content is not consistent across different tumors, these findings collectively suggest that PUFAs, especially arachidonic acid, are important inducers of lipid peroxidation in the TME.

### 5.2 ROS and iron in TME induce lipid peroxidation

ROS encompass a group of unstable oxygen derivatives, including hydrogen peroxide (H_2_O_2_), superoxide anion (O2^−^), hypochlorous acid (HOCl), singlet oxygen (O_2_), and hydroxyl radical (•OH) (129). Numerous physiological processes within cells generate ROS, with mitochondria accounting for approximately 90% of cellular ROS production. Tumor cells, which have a heightened demand for energy to support their proliferation, have been shown to produce elevated levels of ROS compared to normal cells (130). Additionally, hypoxic TME can further stimulate the generation of mitochondrial ROS (131). The increased levels of ROS produced by tumor cells can impact immune cells within the TME. ROS interacts with membrane phospholipids, inducing lipid peroxidation. These phospholipids, rich in PUFAs, are particularly susceptible to ROS-induced damage (132). This process can initiate oxidative stress through a feedback loop triggered by fatty acid peroxidation, leading to alterations in the lipid bilayer of cell membranes and the generation of free radicals (133). Furthermore, PUFA themselves, after reacting with ROS and undergoing lipid peroxidation, can transform into reactive free radicals, thus propagating lipid peroxidation chain reactions (134).

Among the various ROS molecules, the hydroxyl radical (•OH) stands out as one of the most common and potent free radicals (135). This radical is generated through the well-known oxidation process termed the Fenton reaction, where the combination of iron (Fe^2+^) and hydrogen peroxide (H_2_O_2_) gives rise to hydroxyl radicals. In the Fenton reaction, iron (Fe) serves as a catalyst, especially under acidic conditions, to greatly enhance the oxidative potential of H_2_O_2_ (136). Consequently, the presence of iron in TME plays a pivotal role in promoting the production of hydroxyl radicals, which in turn can induce lipid peroxidation.

Within TME, tumor cells exhibit an overexpression of iron importers, such as DMT1 (137), and a downregulation of iron exporter ferroportin (138). This pattern reflects their heightened demand for iron, driven by increased metabolic activities associated with malignancy. In addition, M2 TAM in TME have been identified as a source of iron (139), as they exhibit an iron-releasing phenotype characterized by reduced ferritin and increased ferroportin expression (140). Consequently, in an iron-enriched TME, which fuels tumor progression and metastasis, ample opportunities arise for the generation of ROS and subsequent lipid peroxidation.

### 5.3 Chemotherapy drugs trigger lipid peroxidation in tumor patients

Chemotherapy drugs are a class of medications that are designed to target cancer cells to destroy or inhibit their growth (141). In recent years, accumulating evidence has shown that Several FDA-approved anti-tumor drugs can also trigger lipid peroxidation. For example, sorafenib, a protein kinase inhibitor for the treatment of kidney and liver cancer, induces lipid peroxidation and ferroptosis by suppressing the activity of system Xc^−^ in various types of cancer cells (142–145). In addition, acyl-coA synthetase long chain family member 4 (ACSL4) and glutathione s-transferase zeta 1(GSTZ1) is required for sorafenib-induced lipid peroxidation and ferroptosis (146, 147). Another extensively utilized antitumor agent, cisplatin, has been established to increase lipid peroxide by directly depleting intracellular glutathione S-transferase (GST) and glutathione (GSH) (148–150), and exhibits a synergistic therapeutic effect when combined with erastin (151) and dihydroartemisinin (152). Poly (ADP-ribose) polymerase (PARP) inhibitor olaparib is efficacious in treating ovarian cancer (153). Recent studies showed that olaparib could promote lipid peroxidation and ferroptosis in ovarian cancer by suppressing SLC7A11, and treatment tumor-bearing mice with ferroptosis inducers sulfasalazine markedly sensitized the antitumor efficacy of olaparib (154, 155). These studies imply that chemotherapy drugs induce lipid peroxidation within tumors. Hence, patients undergoing chemotherapy treatment might experience improved responses by augmenting their regimen with additional agents that promote lipid peroxidation and ferroptosis.

### 5.4 GSH plays a crucial role in mitigating lipid peroxidation

Glutathione is a tripeptide molecule composed of three amino acids: cysteine, glycine, and glutamic acid (156). It plays a pivotal role in the cellular antioxidant defense system and is essential for preventing lipid peroxidation caused by free radicals and oxygen (157). During oxidative stress, GSH undergoes conversion by GSH-dependent peroxidases, leading to the formation of GSH disulfide (GSSG) through its interaction with ROS (158). With the involvement of GSH, the GPX4 transforms lipid hydroperoxides into lipid alcohols and this process effectively guards against oxidative lipid damage, lipid peroxidation, and the initiation of ferroptosis (159, 160).

Analysis of tumor samples has shown that certain types of cancer, including breast, lung, ovarian, and head and neck cancers, exhibit notably elevated levels of GSH (161). In breast tumors, GSH levels were more than twice higher than normal breast tissue, and these levels were even higher in lymph node metastases (162). In line with this, another study also reported a significant increase in the levels of GSSG (oxidized glutathione) and total glutathione in breast cancer tissues compared to adjacent cancer-free tissues (163). Similar observations were made in the cases of lung cancer, ovarian cancer, and squamous cell carcinoma (164–167). In addition, patients with elevated GSH levels in ovarian tumor tissues exhibited noticeably lower progression-free survival and overall survival rates compared to those with lower GSH levels (168). Moreover, in the case of tumors with elevated GSH levels, a corresponding decrease in lipid peroxidation was observed (169, 170), indicating that antioxidant molecules contribute to creating a microenvironment conducive to tumor cell proliferation. Nevertheless, certain types of tumors exhibit distinct scenarios, where the GSH levels within tumor tissues are notably lower than in normal tissues, as seen in brain cancers. Interestingly, this reduction of GSH levels in brain tumor tissues corresponded with an increase in lipid peroxidation, as indicated by elevated levels of thiobarbituric acid reactive substances (TBARS) (171). Furthermore, when compared to low-grade brain tumors, high-grade brain tumors exhibited a significant increase in lipid peroxidation along with a reduction in GSH (171). Collectively, these reports indicate there is an inverse correlation between lipid peroxidation and GSH level in tumor tissues, and GSH may serve as guards against lipid peroxidation to promote the development of tumors.

## 6 Therapeutic implications and future perspectives

### 6.1 Regulating lipid peroxidation with small-molecule inducers and inhibitors

Small-molecule compounds are organic chemicals with low molecular weight that readily penetrate cells and influence molecular pathways by targeting vital proteins (172). In recent years, many ferroptosis inducers and inhibitors have been developed by regulating cellular lipid peroxidation across diverse tumor-related models, aiming to assess their impact on tumor cells as well as the immune cells within the tumor microenvironment.

One of the most frequently used ferroptosis inducers in scientific research is RSL3, which directly binds to GPX4 and deactivates its peroxidase activity, leading to the lethal accumulation of lipid peroxidation (173). Other compounds, such as ML162 (174), DPI7 (175), FIN56 (176), and sorafenib (143), also target GPX4 and induce the accumulation of lipid peroxidation, ultimately promoting ferroptosis. Erastin represents a different category of ferroptosis inducers. Its functional mechanism involves the inhibition of the cystine-glutamate antiporter system X_c_− activity (142), which results in cells being unable to synthesize GSH. Consequently, these cells undergo excessive lipid peroxidation and ultimately succumb to ferroptosis. Sorafenib was also reported to inhibit X_c_^−^ activity (177). Though numerous studies have shown that ferroptosis inducers possess promising potential in inhibiting tumor cell growth, their potential effects on impairing the immune cells make the situation complicated (178). For example, it has been reported that CD8^+^ T cells have facilitated tumor ferroptosis by releasing IFN-γ, which disrupts the cystine uptake by tumor cells, triggers lipid peroxidation accumulation, and ultimately results in ferroptotic tumor cell death (6). Considering this perspective, the combination of immunotherapy with ferroptosis induction emerges as a promising strategy to effectively impair tumor growth. However, others also found that CD8^+^ T cells are more sensitive to ferroptosis inducers RSL3 than B16 and MC38 cancer cells (38). Intratumor CD8^+^ T cells underwent lipid peroxidation in lipid laden TME and became dysfunctional (40, 45). As existing lipid peroxidation/ferroptosis inducers have poor selectivity (179), targeting lipid peroxidation to enhance cancer therapy must consider the complex nature of TME.

High-throughput screening efforts have identified ferrostatin-1 and liproxstatin-1 as potent inhibitors of ferroptosis by decreasing lipid peroxidation (180–182). Cui et al. found that within the TME of gastric cancer, L-kynurenine was observed to induce lipid peroxidation and ferroptotic cell death in NK cells, which can be inhibited by ferrostatin-1 (183). In addition, treatment of immunocompetent mice with liproxstatin-1 reversed the suppressive activity of PMN-MDSCs, resulting in reduced tumor progression (112). Furthermore, when combined with immune checkpoint blockade, liproxstatin-1 displayed synergistic effects in suppressing tumor growth (112). These results showed that the use of lipid peroxidation inhibitors also holds promise in enhancing immune response in certain cancers. However, controversial results were found in other reports. In the B16 melanoma model, liproxstatin-1 treatment did not reduce tumor growth but diminished the therapeutic efficacy of immune checkpoints (6). Additionally, liproxstatin-1 administration abolished radiotherapy-induced tumor suppression in ID8 ovarian cancer (184). Therefore, as both immune cells and tumor cells respond to lipid peroxidation-induced stress within TME, cell-specific delivery of lipid peroxidation inducers or inhibitors presents a promising method to enhance cancer therapeutic outcomes.

### 6.2 Synergizing photodynamic therapy with lipid peroxidation as a new strategy for tumor therapy

PDT is a relatively non-invasive anticancer approach that utilizes a photosensitive drug, known as a photosensitizer, along with specific wavelength light irradiation. This combination generates ROS within the tumor tissues, thereby impeding tumor progression (185). PDT can provide the source of active oxygen for the Fenton reaction, which speeds up lipid peroxidation and improves the efficacy of PDT in antitumor therapy (186). Simultaneously, PDT triggers acute inflammation and activates both innate and adaptive immunity, inducing damage-associated molecular patterns (DAMPs) (187, 188). Considering these aspects collectively, it becomes reasonable to explore the combination of PDT with lipid peroxidation-related agents as a strategy to activate immune responses and eliminate tumor responses (189).

Nanoparticle materials have been introduced to selectively deliver lipid peroxidation inducer and combined PDT together to stimulate the immune response (186), leading to the elimination of tumor cells. Xu et al. devised a nano-platform wherein hemoglobin is conjugated with the photosensitizer chlorin e6 to construct a 2-in-1 nanoplatform with sorafenib loaded (190). This nanoplatform effectively merges oxygen-enhanced PDT, clinical chemotherapy drugs, and lipid peroxidation-induced ferroptosis to target tumor cells. Apart from sorafenib’s direct role in inducing the lethal accumulation of lipid peroxidation, PDT intensifies the ferroptosis process by recruiting immune cells that release IFN-γ, thereby sensitizing the tumor cells to ferroptosis (190). In addition, another group established a cytotoxic T-cell-inspired oncolytic system to precisely lyse cancer cells by NIR-light-controlled lipid peroxidation, which specifically induces lipid peroxidation in tumor cells and enhances T-cell-based antitumor ability (191).

### 6.3 Challenges in targeting lipid peroxidation for cancer therapy

Lipid peroxidation varies significantly among different types of tumors, individuals, and even in response to various therapeutic strategies. Tumor and immune cells exhibit distinct reactions in exposure to the same levels of lipid peroxidation. Therefore, nanoparticles that selectively deliver lipid peroxidation inducer to tumor cells may be a promising way to enhance cancer therapy. For example, nanoparticles co-delivering dihydroartemisinin and lipid peroxidation inducer RSL3 have been shown to efficiently induce ferroptosis in tumor cells of pancreatic ductal adenocarcinoma, melanoma, and metastatic breast tumors, and trigger T cell-based antitumor immunity (192, 193). Notably, the co-delivery system has also demonstrated a synergistic effect in inhibiting pancreatic ductal adenocarcinoma tumor growth when combined with PD-L1 blockade therapy (192). However, the safety and efficiency of this co-delivery system are still under investigation.

## 7 Discussion

**Figure 4:**
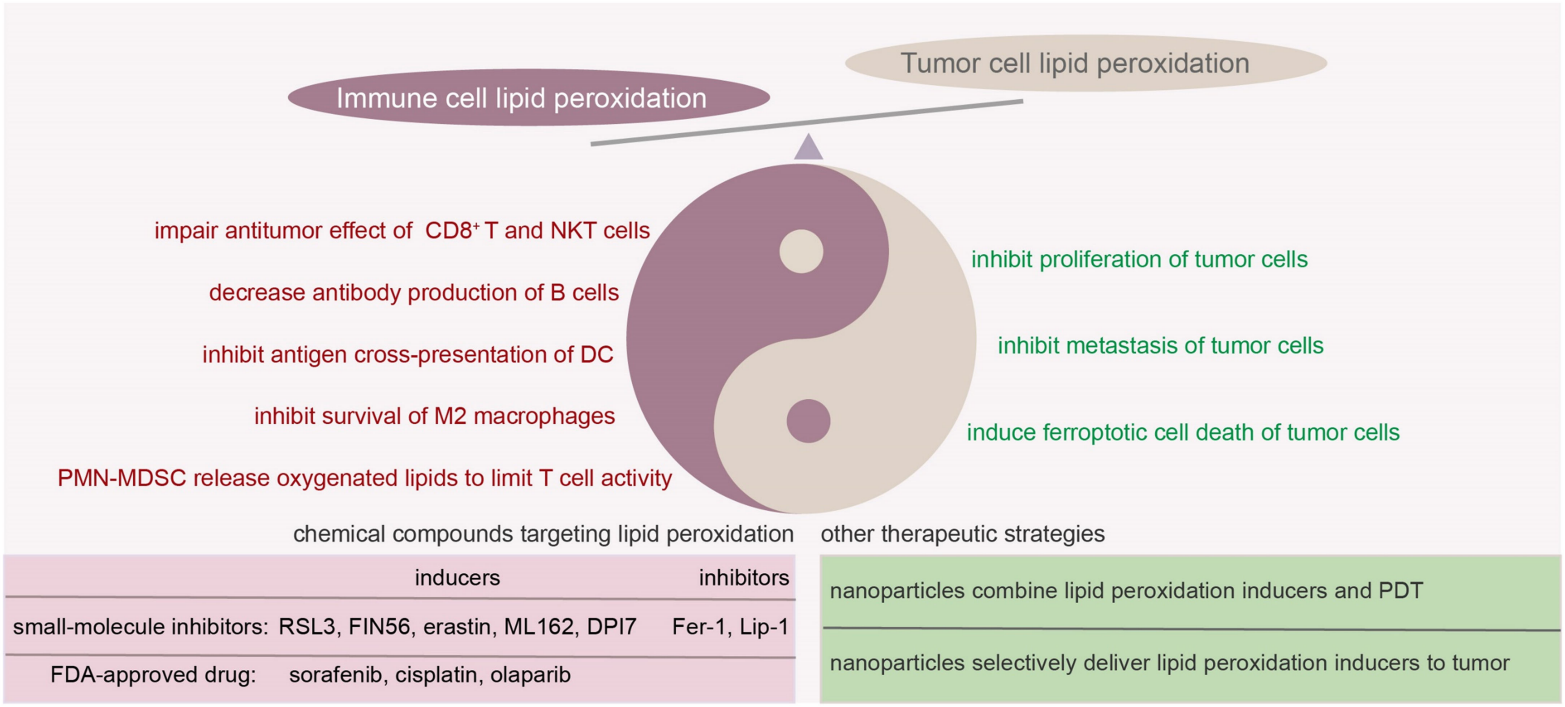
Double edged sword of lipid peroxidation in tumor microenvironment and potential strategies to target lipid peroxidation. In TME, tumor cells and immune cells respond differently when exposed to the same levels of lipid peroxidation. In the case of tumor cells, an increase in lipid peroxidation often aids in inhibiting tumor progression. For certain immune cells, overwhelming lipid peroxidation might potentially weaken their immune responses. Several small-molecule compounds and other therapeutical strategies were raised to target lipid peroxidation. When considering the regulation of lipid peroxidation to inhibit tumor progression, it’s essential to account for both tumor cells and immune cells. The scenarios can become quite intricate and necessitate case-by-case evaluation. Fer-1, ferrostatin-1; Lip-1, liproxstatin-1; PDT, photodynamic therapy.

Although it has been demonstrated that lipid metabolism plays important roles in modulating immune cell function and targeting lipid metabolism is a novel strategy to enhance antitumor immunity, the role and clinical potential of lipid peroxidation in immune cells remain unclear. Since the discovery that therapy-resistant cancer cells exhibited sensitivity to ferroptosis (194–196), modulating cellular lipid peroxidation and induction of ferroptosis in cancer cells have been considered a potential therapeutic strategy. However, in most of these studies, the role of lipid peroxidation in the immune system was not fully considered as these studies were based on xenograft mouse tumor models. In fact, as a double-edge sword, lipid peroxidation not only affects the survival of tumor cells, but also influences the function of the immune cells (**Figure 4**). In this review, we summarize the effect of lipid peroxidation on the function of various immune cells in TME and emphasize the distinct sensitivities of different immune cells to tumoral lipid peroxides. These studies provide new strategies to enhance the efficacy of immunotherapy by targeting lipid peroxidation in immune cells in a cell-specific manner. For example, ex vivo pharmacologic or genetic reprogramming of CD8^+^ T cell lipid peroxidation prior to adoptive cellular therapy was able to enhance their effector function or longevity *in vivo (* 40, 45, 49).

Current studies have provided new insights into the molecular mechanisms of lipid peroxidation in regulating immune cell function in TME. The interaction among immune cells, tumor cells, and nutrients in TME plays an important role in the induction of lipid peroxidation in the cells, tumor development, and tumor immunity. However, the detailed underlying mechanisms driving different lipid peroxidation and sensitivity in different cell subsets await further exploration. Future work should address the specific source of lipid peroxides in TME, the key molecules in driving lipid peroxidation to ferroptosis, as well as lipid peroxidation interdependence of immune cells and cancer cells in TME. It is important to understand whether and by what mechanisms immune cells can obtain maximum antitumor ability by targeting lipid peroxidation. Undoubtably, a comprehensive understanding of lipid peroxidation in both tumor cells and immune cells within TME will provide novel and feasible therapeutic strategies for cancer.

## Author contributions

QY: Conceptualization, Writing – review & editing. LX: Conceptualization, Writing – original draft, Writing – review & editing. MX: Writing – original draft. CZ: Writing – original draft. QG: Writing – review & editing.
